# Participation needs of older adults having disabilities and receiving home care: met needs mainly concern daily activities, while unmet needs mostly involve social activities

**DOI:** 10.1186/s12877-015-0077-1

**Published:** 2015-08-01

**Authors:** Pier-Luc Turcotte, Nadine Larivière, Johanne Desrosiers, Philippe Voyer, Nathalie Champoux, Hélène Carbonneau, Annie Carrier, Mélanie Levasseur

**Affiliations:** 1 School of Rehabilitation, Faculty of Medicine and Health Sciences, Université de Sherbrooke, Sherbrooke, Québec Canada; 2 Research Centre on Aging, Health and Social Services Centre-University Institute of Geriatrics of Sherbrooke, Sherbrooke, Québec Canada; 3 Faculty of Nursing Sciences, Université Laval, Québec City, Québec Canada; 4 Québec Centre for Excellence in Aging, Québec City, Québec Canada; 5 Department of Family Medicine, Université de Montréal, Montréal, Québec Canada; 6 Research Centre of the University Institute of Geriatrics of Montreal, Montréal, Québec Canada; 7 Department of Leisure, Culture and Tourism Studies, Université du Québec à Trois-Rivières, Trois-Rivières, Québec Canada

**Keywords:** Needs Assessment, Home Care Services, Social and Healthcare Services, Participation, Daily Activities, Social Activities, Older Adults, Caregivers, Healthcare Providers

## Abstract

**Background:**

Participation is a key determinant of successful aging and enables older adults to stay in their homes and be integrated into the community. Assessing participation needs involves identifying restrictions in the accomplishment of daily and social activities. Although meeting participation needs involves older adults, their caregivers and healthcare providers, little is known about their respective viewpoints. This study thus explored the participation needs of older adults having disabilities as perceived by the older adults themselves, their caregivers and healthcare providers.

**Methods:**

A qualitative multiple case study consisted of conducting 33 semi-structured interviews in eleven triads, each composed of an older adult, his/her caregiver and a healthcare provider recruited in a Health and Social Services Centre (HSSC) in Québec, Canada. Interview transcripts and reviews of clinical records were analyzed using content analysis and descriptive statistics based on thematic saliency analysis methods.

**Results:**

Aged 66 to 88 years, five older adults had physical disabilities, five had mild cognitive impairment and one had psychological problems, leading to moderate to severe functional decline. Caregivers and healthcare providers were mainly women, respectively retired spouses and various professionals with four to 32 years of clinical experience. Participation needs reported by each triad included all domains of participation. Needs related to daily activities, such as personal care, nutrition, and housing, were generally met. Regarding social activities, few needs were met by various resources in the community and were generally limited to personal responsibilities, including making decisions and managing budgets, and some community life activities, such as going shopping. Unmet needs were mainly related to social activities, involving leisure, other community life activities and interpersonal relationships, and some daily activities, including fitness and mobility.

**Conclusions:**

This study highlights the complexity of older adults’ participation needs, involving daily as well as social activities. Properly assessing and addressing these needs is thus necessary to improve older adults’ health and well-being. Discrepancies in the various actors’ perceptions of participation needs must be further explored. Additional research would help better understand how to optimize the contribution of community organizations and caregivers.

## Background

Aging of populations and increasing prevalence of chronic diseases are two major public health concerns of developed countries [1]. By 2031, 25 % of Québec’s population in Canada will be aged 65 years or over [2], the most significant demographic change ever seen in this province. While getting older, 42 % of adults will experience disabilities [3] that could restrict their participation in daily and social activities [4]. Aging can also be associated with occurrence of chronic diseases, such as heart diseases, cancer or stroke [5] and lower quality of life. Furthermore, chronic diseases are the leading causes of death [6] and of activity limitation [5] and tend to increase [7]. In consequence, aging and disabilities are two important challenges that will rise health expenditure and diminish accessibility to healthcare systems [1]. To face these modern challenges, health policies should enable healthy aging by shifting from a “curative” to a “preventive” paradigm [5]. Such a preventive approach is fully embodied in successful aging models, which encompass avoiding disease and disability, high physical and cognitive function, and active engagement with life [8]. Focusing on more positive sides of aging, successful aging goes beyond potential, and involves activity in a multidimensional manner.

To meet the growing needs of older adults having disabilities, social and healthcare systems require innovative and efficient interventions aimed at optimizing their participation in the community, an important modifiable determinant of health and successful aging [9]. Although there is no consensus regarding its definition, the concept of participation is included in two well-known models: the Human Development Model-Disability Creation Process (HDM-DCP) [10] and the International Classification of Functioning, Disability and Health (ICF) [11]. According to the HDM-DCP, participation refers to the accomplishment of daily and social activities valued by a person or his/her sociocultural context. In that model, daily activities include nutrition, fitness, personal care, communication, housing and mobility; while social activities refer to responsibilities, interpersonal relationships, community life, employment, education and leisure (Table 1). Even though this model was not specifically developed in the context of healthy and successful aging, the HDM-DCP provides a clear conceptualization of a person’s life habits and their interactions with other determinants of health and well-being. Moreover, based on a conceptual comparison with the ICF [12], and as the input of people having disabilities was considered throughout its development, the HDM-DCP was found to encapsulate the concept of participation and especially social participation more accurately. Through a bi-directional interaction between an individual’s characteristics (personal factors) and his/her life context (environmental factors), many links can be made between participation and health promotion models. Accordingly, creating supportive environments, building community participation and developing personal skills are some of the core concepts of the Ottawa Charter for Health Promotion [13]. More specifically, social activities correspond to the involvement of a person in activities providing interactions with others [14], consistent with a person’s life expectations [15], and contribute to an increased perception of health [16] and delayed functional decline [17]. Furthermore, being involved in social activities, such as leisure, community life and interpersonal relationships, was shown to be more associated with quality of life than pursuing daily activities [18]. To improve health and well-being, interventions should target an optimal participation by increasing physical, cognitive or psychological abilities, adapting the activities to enable their accomplishment, or modifying the environment to be safe and supportive [19]. As such, participation is a central component of an individual’s rehabilitation towards active engagement with life [11], often targeted by health interventions [20].

**Table 1:** Dimensions and definitions of participation in the Human Development Model-Disability Creation Process (Fougeyrollas, 2010)

Dimensions	Definitions
Daily activities	
1. Nutrition	Habits related to food consumption (choosing foods, food preparation, preparing meals, etc.)
2. Fitness	Habits related to fitness of body and mind (sleep, naps, physical and mental fitness, etc.)
3. Personal care	Habits related to physical well-being (hygiene, excretory hygiene, dressing, health care)
4. Communication	Habits that enable a person to exchange messages with others (oral, sign, and written communication, telecommunication)
5. Housing	Habits related to individual’s place of residence (lodging, home maintenance, use of furnishing and other household appliances)
6. Mobility	Habits related to mobility over short and long distances with or without means of transportation (using means of transportation, generally within the immediate environment, such as walking and using a car.)
Social activities	
7. Responsibilities	Habits related to taking up responsibilities (financial, civil and family responsibilities, such as preparing and respecting a budget, respect for others, civic responsibility and the care of a person, such as children and spouses.)
8. Interpersonal relationships	Habits concerning relationships with others (sexual activity, affective relationships)
9. Community life	Habits related to activities within the community (participating in social organizations, such as citizen and social clubs, and in spiritual life and religious practice)
10. Education	Habits related to individual psychomotor, intellectual, social and cultural development (participating in a training program, etc.)
11. Employment	Habits related to the principal occupation of the adult individual; usually a paid occupation, but also volunteering
12. Leisure	Habits related to recreational activities or others practiced during an individual’s free time and within a pleasurable context (sports and games, arts and culture, and socio-recreational activities)

To foster older adults’ participation, healthcare providers must assess their needs and deliver associated services. By addressing gaps between current and desired conditions [21], participation needs assessment refers to the identification of restricted accomplishment of daily and social activities [10]. In Québec (Canada), social and healthcare systems have the legal obligation to realize such an assessment in order to meet the needs of the whole population in their territory (Act Respecting Health Services and Social Services [LSSSS], art. 1). Currently, in each of the 94 local territories of the province of Québec, Health and Social Services Centres (HSSCs) are the authorities responsible for offering accessible, integrated, and continuous high-quality services through health promotion and prevention, rehabilitation and social integration [20]. Each HSSC operates a local community services centre, a residential and long-term care facility and, where applicable, a general or specialized acute care hospital. As with other long-term and primary healthcare settings in various developed countries [22, 23], to meet the growing needs of their population, HSSCs must establish partnerships with other resources, including informal caregivers, private enterprises, community organizations and social enterprises [24]. To properly inform and coordinate their interventions, healthcare professionals must assess the needs of older adults considering the whole range of participation opportunities, including daily as well as social activities. To do so, needs assessment should not only be client-centred, but also include the client’s personal and environmental aspects, organizational factors as well as those related to the service providers [25].

Although important, little is known about the participation needs of older adults having disabilities and receiving home care. A study conducted among users of a HSSC home care program (*n* = 8 434) revealed that only 8 % of reported needs in daily activities were satisfied [26]. Moreover, in another study involving older adults who had a stroke and their caregivers, psychological needs, such as coping with health conditions and receiving emotional support, were reported to be a priority, but remained mainly unmet [27]. A qualitative study that aimed to explore perceptions regarding health issues and that was conducted in various Australian health settings showed that persons with chronic illness, as well as their caregivers and health professionals agreed about the complexity of managing multiple health conditions and their impacts on their daily life [28]. In that study, when patients and caregivers were mainly concerned about their personal experiences and challenges, healthcare professionals mainly focused on their own resources to explain the patients’ reality. Another Sweden qualitative study about the experiences in relation to the participation of older adults living at home and caregivers found that older adults’ goals included performing meaningful activities and routines accomplished independently from other people [29]. However, these studies mainly addressed daily activities and did not provide an in-depth exploration of the met and unmet needs of participation in daily and social activities. The present study thus aimed to explore participation needs among older adults having disabilities as perceived by older adults themselves, their caregivers and the HSSC healthcare providers.

## Methods

### Design

Part of a larger research initiative of the same research team, this qualitative study was conducted from January 2011 to February 2012 using a multiple case study design [30]. This allowed to provide an in-depth analysis and description of a social phenomenon, in the present case, of older adults’ more in-depth met and unmet participation needs. The Eastern Townships’ HSSCs Ethics Research Committee approved this study (2011-261, 2010-36/Levasseur).

### Participants

Data were collected with three types of participants: 1) older adults aged 65 years and older receiving home care services from a HSSC in the province of Québec (*n* = 11), 2) their principal caregivers (*n* = 11), and 3) healthcare providers in the home care (*n* = 10) and the mental health (*n* = 1) programs of the same HSSC. Participants were recruited by two research assistants using a convenience strategy. A list of potential older adults was provided to the research team by the HSSC clinical coordinators based on our eligibility criteria. To be eligible, they had to understand and speak French. In addition, older adults had to: 1) have a score of 15 or more on the Functional Autonomy Measurement System (*Système de mesure de l’autonomie fonctionnelle* [SMAF]) [31], indicating a moderate to severe loss of functional autonomy; 2) live in a conventional or a residential home for independent or semi-independent older adults, 3) receive home care services from a HSSC, and 4) have a caregiver who agreed to participate to the study. Since their experiences and needs were different and could have influenced their capacity to participate to the data collection, older adults unable to consent, presenting significant difficulties in communication or those in palliative care were excluded. Caregivers had to regularly look after the older adults, and healthcare providers had to be the main clinician working with the older adults at the time of the study or have been actively involved in the provision of services in the previous six months. All participants gave their informed written consent prior to the interviews.

### Data collection and instruments

Conducted separately with the older adult, the caregiver and the healthcare provider in the setting of their choice, semi-structured interviews were realized by a research assistant. Using a semi-structured interview guide developed for the purposes of this study, interviews lasted between 60 and 90 min. General and specific questions from the interview guide for each domain of participation are given in Table 2. A follow-up telephone interview was done when needed to clarify information.

**Table 2:** Semi-structured interview guide developed for the purposes of this study

General questions	Tell me about the things you usually do in a typical day. Since onset of disease or disability, what changes have occurred in the accomplishment of your usual activities? Tell me about your social activities in the community, i.e. outside your home. What are your requirements in relation to your activities? What help do you receive to carry out your activities, what resources do you use to conduct your affairs?
Specific questions	a) According to your experience, what are your needs related to (ask question for each activity)? Which activities related to (the current domain of participation) do you find more difficult to do? What is more difficult?
	b) Are there some activities related to (the current domain of participation) you decided to do differently because of your disabilities? Do you do these activities as often as you would like? How do you feel about the fact that you do these activities differently or less often?
	c) Are there some activities related to (the current domain of participation) that you decided to stop doing because of your disabilities? Are there other activities related to (the current domain of participation) that you would like to do but that you don’t do? How do you feel about the fact that you no longer do these activities?
	d) What help or support do you use to do your activities related to (the current domain of participation), for example, equipment, taking more time, or using human help to do the activity? What do you think about this help you receive?
	e) What other resources or services do you receive related to (the current domain of participation)?
	f) To what extent do these resources or services you receive meet your needs for your activities related to (the current domain of participation)? Could they be better adapted to your needs? How? What resources or services could be put in place to help you do your activities related to (the current domain of participation)? What other resources or services would you need to do these activities that you would like to do but that you don’t do?

Usual sociodemographic and clinical data were collected for all participants, involving their age, gender, education, and professional status. For older adults and caregivers, self-perceived health was assessed using a scale from: excellent, good, fair, to poor. This scale is particularly suitable to describe the health status of older people [32]. Residential status, income, type of disability and health problems, and time since onset of health problems were considered for older adults, while relationship with older adult and experience were collected for caregiver and healthcare provider respectively.

Records contained in the older adults’ clinical files were reviewed with a data extraction grid, which included the participants’ clinical data, i.e. functional autonomy, health interventions, and needs, and professional notes that allowed enriching the data related to participation needs and their fulfillment. Functional autonomy, as measured with the SMAF [31], was also extracted from the clinical files. The SMAF includes 29 functions covering five domains of activity (number of items): activities of daily living (7), mobility (6), communication (3), mental functions (5), and instrumental activities of daily living (8). Interview guides and data extraction grid were reviewed by an external group of experts in qualitative research and evolved during data collection [33]. Through an iterative process, data collection and preliminary analysis were conducted simultaneously. When the analysis did not provide any new elements regarding the aim of the study, indicating a certain level of saturation, data collection was stopped. To enhance the trustworthiness of the findings, all interviews were audiotaped and transcribed [34]. Furthermore, multiple sources of information (interviews, clinical record reviews, sociodemographic questionnaires) were used to perform data triangulation and provide a holistic insight into the situation.

### Data analysis

As it plays a valuable role in health services research [35], a combination of qualitative and quantitative methods as described by Miles and Huberman (2014) and Yin (2012) was used [30, 36]. As suggested by Yin (2012), this method allowed addressing rival explanations of our findings [30] and promote data triangulation. A qualitative content analysis [36] was performed to identify needs and their fulfilment using a coding manual based on the interview guides and data extraction grids. Needs were identified by explicit gaps between desired and current level of participation in daily and social activities of each older adult [21]. Codes were developed, discussed and revised during team meetings with the two first and last authors (PLT, NL, AC and ML) which ensured rigour through constant monitoring of analysis and interpretation [37]. Interviews transcripts and clinical records for each triad were analyzed separately and syntheses were then written following analyses of each case. Analyzes were performed using QSR NVivo software (version 9.0) and supported with a coding manual as well as Microsoft Word.

Based on thematic saliency analysis methods [38], themes and content emerging of qualitative data analysis were quantified to inform not only about categories of meaningful data segments, but also about their recurrence. As such, the quantitative needs were indicative only of their magnitude in each domain of participation. Categories included components of the HDM-DCP [10] in which data were gathered to identify whether older adults had: 1) perceived needs, 2) met needs, or 3) unmet needs. To ensure reliability of the findings and allow comparison between the study participants, as they were not addressed by the majority of them, the education and employment domains were not considered for this quantitative part of analysis. ‘Perceived’ needs were identified when at least one of the participants mentioned that the older adult had difficulties or was restricted in the accomplishment of a specific activity. For each activity, needs were considered as ‘met’ when a difficulty was compensated by any resource, or ‘unmet’ when a difficulty remained unsatisfied. Resources could involve support received by informal caregivers, community organizations, private resources or HSSC healthcare providers [24]. Each participant’s perceptions of needs were first categorized separately. Then, for each triad, if participants mentioned that they perceived a need in that older adult’s participation and either the need was met or unmet, this was respectively identified as: 1) having needs, 2) needs being met, or 3) needs being unmet. These needs were described, and quotes were selected to represent all participants’ perspectives and indicate most recurring and important themes. The need and the state of its fulfilment (met or unmet) were reported even if the need was identified by only one of the three participants in a triad. This meant that, needs, especially unmet ones, were not underestimated, which could have critical consequences for health and well-being [39]. Finally, sociodemographic characteristics and quantitative needs were reported using median (Md), interquartile range (I.R.), mean and standard deviation (S.D.) for continuous variables, and frequency and percentage for categorical variables.

## Results

Older adults [A] included five women and six men aged 66 to 88 years old, where five had physical disabilities, five had mild cognitive impairments and one had psychological difficulties (Table 3). The majority were retired, had 11 or less years of schooling, had two or more health problems and perceived their health as good to fair. According to their SMAF, they had most frequently difficulties in their instrumental activities of daily living (Table 3). The caregivers [C] were nine women and two men aged 55 to 90 years old, including six spouses, were mostly retired and had a good to excellent self-perceived health. Healthcare providers [H] were mostly women, with 15 to 16 years of schooling and 4 to 32 years of clinical experience with older adults (Table 3). In addition to having been or currently being followed by a home or a mental healthcare provider, four older adults received help with bathing, three attended to a day centre and two benefited from a voucher program (direct allocation for personal assistance services or long-term domestic help at home).

**Table 3:** Characteristics of participants (*n* = 33)

	Older adults (*n* = 11)		Caregivers (*n* = 11)		Healthcare providers (*n* = 11)	
Continuous variables	Md (I.R.)	Mean (S.D.)	Md (I.R.)	Mean (S.D.)	Md (I.R.)	Mean (S.D.)
Age (years)	78.0 (7.0)	77.9 (7.7)	66.0 (6.0)	70.2 (12.2)	44.0 (3.0)	44.5 (6.9)
Time since onset of health problems (years)	10.0 (6.5)	8.9 (5.9)				
Functional autonomy (SMAF)						
ADL (/21)	6.0 (3.5)	7.3 (3.9)				
Mobility (/18)	4.0 (2.3)	5.2 (4.5)				
Communication (/9)	0 (1.0)	0.7 (1.0)				
Mental functions (/15)	3.0 (1.5)	2.8 (2.3)				
IADL (/24)	17.0 (1.3)	16.1 (3.5)				
Total (/87)	29.5 (9.3)	32.1 (10.0)				
Clinical experience (years)					18.0 (5.5)	17.8 (9.4)
Categorical variables	*n* (%)		*n* (%)		*n* (%)	
Gender (women)	5 (45.5)		9 (81.8)		7 (63.6)	
Education (years)						
• Elementary (1–6)	3 (27.3)		1 (9.1)			
• High School (7–11)	4 (36.4)		5 (45.5)			
• College/professional degree (12–14)	3 (27.3)		2 (18.2)		2 (18.2)	
• Bachelors (15–16)			2 (18.2)		7 (63.6)	
• Masters/Doctorate (>17)			1 (9.1)		2 (18.2)	
Type of disability						
• Physical	5 (45.5)					
• Cognitive	5 (45.5)					
• Psychological	1 (9.1)					
Two or more health problems (yes)	9 (81.8)					
Health problems						
• Musculoskeletal	6 (54.5)					
• Neurological	6 (54.5)					
• Cardiovascular	4 (36.4)					
• Digestive	4 (36.4)					
• Mental	3 (27.3)					
• Pulmonary	2 (18.2)					
• Visual	1 (9.1)					
• Auditory	1 (9.1)					
Residential status						
• Owner	6 (54.5)					
• Tenant	3 (27.3)					
• Private residence	2 (18.2)					
Income (Can $)						
• < 15 000	3 (27.3)					
• 15 001–25 000	3 (27.3)					
• > 25 000	1 (9.1)					
*Missing data*	4 (36.4)					
Self-perceived health						
• Excellent	1 (9.1)		5 (45.5)			
• Good	6 (54.5)		4 (36.4)			
• Fair	3 (27.3)		2 (18.2)			
• Poor	1 (9.1)		0 (0)			
Relation with older adult						
• Spouse			6 (54.5)			
• Children			3 (27.3)			
• Sibling			2 (18.2)			
Professional status/title						
• Retired	11 (100.0)		8 (72.7)			
• Unemployed			1 (9.1)			
• Full-time worker			1 (9.1)			
• Part-time worker			1 (9.1)			
• Occupational therapist					1 (9.1)	
• Case manager					4 (36.4)	
• Respiratory therapist					1 (9.1)	
• Nurse					2 (18.2)	
• Medical doctor					1 (9.1)	
• Social worker					2 (18.2)	

*Md* median, *IR* interquartile range, *SD* standard deviation, *SMAF* Functional Autonomy Measurement System, *ADL* activities of daily living, *IADL* instrumental ADL

### Perceived needs

Almost every older adult had participation needs identified for at least one daily and social activities (Table 4). Regarding daily activities, five to eleven older adults had at least one specific perceived need in each domain. Among the eleven triads, a median of ten and eleven participants reported needs respectively in nutrition or fitness, and personal care, housing or mobility (Table 4). One participant who lived with Parkinson disease, talking about his wife preparing his meals, mentioned: “I limit myself a lot. […] I could do more but she’s always there ahead of me so I don’t do it” [A10]. Many older adults had a hard time sleeping, as illustrated by this healthcare professional: “The times he said he didn’t sleep well were because he was a bit more worried” [H04]. Fitness, particularly physical exercises, was an issue for the many of participants, as recognized by this man who had chronic obstructive pulmonary disease: “I should do more because I notice that when I don’t, I go downhill. I’m really disheartened about it” [A04]. Also, cognitive fitness stimulation was often required, as acknowledged by this healthcare professional: “She would benefit from the day centre, the stimulation of being with people; we know all the advantages of being with other people” [H03]. Regarding personal care, including body hygiene, dressing and taking medication, all older adults had perceived needs, sometimes because of environmental barriers or personal issues: “She can’t get into the bath by herself because of her knee. She has fallen in the past and now she’s afraid” [C05]. The majority of older adults had difficulties in expressing their needs, as illustrated by this daughter who had to read in her mother’s mind: “My mother never complains, we have to guess” [C03]. Choosing a home where to live in the event older participants lost their caregiver or home care support generated worries. For example, this older woman questioned herself about her future home without her husband: “He’s with me all the time. If he passes away—he’s 90 years old—what will I do? I shouldn’t think about that. I’m 90 years old. I’d have to go into a home. And I haven’t chosen one yet” [A01]. Losing his driver’s license made transportation a major issue for this older man: “[…] losing my freedom. Before I could go out anytime and do anything I wanted: go to a show, visit a museum, whatever. […] I can’t do that anymore” [A08].

**Table 4:** Participation needs and their fulfillment identified by any one of the following participants: older adult, caregiver or healthcare provider (*n* = 11)

	Perceived needs			Met needs			Unmet needs		
	*n* (%)^a^	Md (I.R.)	Mean (S.D.)	*n* (%)^a^	Md (I.R.)	Mean (S.D.)	*n* (%)^a^	Md (I.R.)	Mean (S.D.)
Daily activities^b^		10 (2.0)	9 (1.9)		3 (2.0)	4 (3.1)		6 (3.0)	5 (3.0)
Nutrition		10 (2.3)	9 (2.1)		5 (3.8)	5 (4.1)		4 (1.8)	4 (2.9)
Preparing a meal	11 (100.0)			8 (72.7)			3 (27.3)		
Choosing a meal	10 (90.9)			8 (80.0)			2 (20.0)		
Doing groceries	11 (100.0)			10 (90.9)			1 (9.1)		
Eating a meal	7 (63.6)			2 (28.6)			5 (71.4)		
Going to the restaurant	10 (90.9)			1 (10.0)			9 (90.0)		
Following a diet	6 (54.6)			1 (16.7)			5 (83.3)		
Fitness		10 (0.5)	10 (0.6)		0 (0.0)	1 (1.7)		9 (1.0)	9 (1.5)
Sleeping	10 (90.9)			3 (30.0)			7 (70.0)		
Doing physical exercises	10 (90.9)			0 (0.0)			10 (100.0)		
Having cognitive stimulation	9 (81.8)			0 (0.0)			9 (100.0)		
Personal care		11 (1.3)	10 (1.3)		8 (2.0)	6 (2.4)		4 (2.0)	4 (3.0)
Maintaining body hygiene	11 (100.0)			5 (46.4)			6 (54.6)		
Performing foot care	9 (81.8)			8 (88.9)			1 (11.1)		
Doing laundry	8 (72.7)			8 (100.0)			0 (0.0)		
Taking medication	11 (100.0)			8 (72.7)			3 (27.3)		
Dressing	11 (100.0)			7 (63.6)			4 (36.4)		
Using the toilet	10 (90.9)			2 (20.0)			8 (80.0)		
Communication		7 (0.5)	7 (1.8)		2 (0.5)	2 (1.5)		5 (1.5)	5 (2.9)
Writing	7 (63.6)			1 (14.3)			6 (85.7)		
Communicating verbally	8 (72.7)			0 (0.0)			8 (100.0)		
Expressing needs	10 (90.9)			1 (10.0)			9 (90.0)		
Using telephone	6 (54.6)			3 (50.0)			3 (50.0)		
Using computer	5 (45.5)			2 (40.0)			3 (60.0)		
Using television	6 (54.6)			4 (66.7)			2 (33.3)		
Housing		11 (1.0)	10 (1.2)		5 (2.5)	4 (3.6)		6 (1.0)	6 (2.5)
Maintaining home	11 (100.0)			7 (63.3)			4 (36.4)		
Doing domestic chores	11 (100.0)			5 (45.4)			6 (54.6)		
Choosing a home	9 (81.8)			0 (0.0)			9 (100.0)		
Mobility		11 (1.0)	10 (1.2)		2 (0.0)	3 (1.2)		7 (0.0)	8 (1.2)
Moving outside the home	11 (100.0)			2 (18.2)			9 (81.8)		
Moving inside the home	9 (81.8)			2 (22.2)			7 (77.8)		
Transporting oneself	11 (100.0)			4 (36.7)			7 (63.6)		
Social activities^b^		10 (1.0)	9 (2.1)		1 (1.0)	2 (2.7)		6 (2.0)	7 (2.9)
Responsibilities		10 (1.5)	9 (2.6)		5 (1.8)	5 (2.6)		4 (0.0)	4 (0.5)
Assuming finances	10 (90.9)			6 (60.0)			4 (40.0)		
Assuming personal choices	11 (100.0)			7 (63.6)			4 (36.4)		
Assuming family responsibilities	5 (45.5)			1 (20.0)			4 (80.0)		
Assuming civil responsibilities	9 (81.8)			4 (44.4)			5 (55.6)		
Interpersonal relationships		9 (1.3)	8 (3.0)		1 (0.5)	1 (1.4)		7 (1.5)	7 (3.0)
Having relations with partner	9 (81.8)			3 (33.3)			6 (66.7)		
Having relations with family	9 (81.8)			1 (11.1)			8 (88.9)		
Having neighborhood relations	4 (36.4)			0 (0.0)			4 (100.0)		
Having relations with friends	11 (100.0)			0 (0.0)			11 (100.0)		
Community life		11 (0.8)	10 (1.0)		1 (1.0)	2 (3.3)		9 (2.3)	8 (3.2)
Being involved in collectivity	11 (100.0)			0 (0.0)			11 (100.0)		
Going shopping	11 (100.0)			7 (63.6)			4 (36.4)		
Doing group activities	10 (90.9)			0 (0.0)			10 (100.0)		
Having religious activities	9 (81.8)			2 (22.2)			7 (77.8)		
Leisure		10 (0.0)	10 (0.6)		0 (0.0)	1 (2.3)		10 (2.0)	9 (2.6)
Pursuing leisure activities	11 (100.0)			0 (0.0)			11 (100.0)		
Having relaxing activities	10 (90.9)			4 (40.0)			6 (60.0)		
Being involved in sports	10 (90.9)			0 (0.0)			10 (100.0)		

*Md* median, *IR* interquartile range, *SD* standard deviation

^a^Presentation of descriptive statistics were indicative only of the participation needs emerging of qualitative content analysis

^b^Although the same participant could have more than one need for each dimension, each participant appeared only once in the total of the dimension. Totals of overall perceived, met and unmet needs could thus not go above eleven

Concerning social activities, nine to eleven participants of the triads perceived needs in each domain principally in community life (11), leisure (10) and responsibilities (10) (Table 3). Most of the time personal and financial responsibilities required an intervention. This clinical file excerpt described this older woman’s difficulties with managing her money, compensated by her daughter: “She’s able to write a cheque but needs help with her banking. Her daughter has a bank power of attorney and goes to the bank to get money for her” [File 05]. According to this daughter, supporting her mother in having relationships with friends was important: “It’s clear that having a friend […] would help” [C03]. The need to take part in group activities was frequently mentioned by participants, including from this health professional: “Maybe she [the older adult] would need more activities inside her residence. Something realistic, that would interest her… improve her general well-being and get her out of her apartment. Social contacts, also, would be interesting” [H11]. As reported in this man’s clinical file, despite having a limited physical endurance, older adults still wanted to be engaged in sports activities: “He would like to go fishing in a few weeks” [File 04]. Specifically, older adults needed stimulation to stay involved in leisure, as this nurse specified: “She is apathetic, she needs to be stimulated to do activities. We need to find out what her main interests are and check with her if there are any interesting activities in the residence. Or even outside, going out with paratransit” [H09].

### Met needs

Fulfilled needs were mainly about personal care, nutrition, housing, some responsibilities and fewer community life activities (Table 4). Even though needs were identified in both daily and social activities, most fulfilled ones concerned daily and less frequently social activities. Specifically for daily activities, personal care was the most fulfilled domain with a median of eight older adults having met needs over eleven triads, followed by nutrition and housing. Needs in meal preparation were mostly responded by external human resources as noticed here: “The couple hires a cook to prepare their meals and the husband reheats them. The caregiver prepares side dishes. She [wife] says she is satisfied with her meals” [File 01]. As he had difficulties with his mobility, this older man’s needs regarding doing the groceries were compensated by his caregiver: “I used to go with my wife to do the grocery shopping. I helped her. But now she does it all because I don’t take any chances. […] in case I fall and hit my head” [A07]. Needs related to personal care, such as hygiene or dressing, as well as housekeeping were the main focus of healthcare professionals. According to this case manager, optimizing the older person’s participation in activities involved to pay more attention to basic activities of daily living rather than instrumental activities: “I don’t see why we would ask him to expend more energy on housekeeping, at the risk of being exhausted, so that he doesn’t have any energy or physical capacities to take care of himself. Activities of daily living are what we must focus on for autonomy and participation” [H08]. To compensate difficulties and meet needs in housekeeping, services were mainly allocated by caregivers, community resources or HSSC, as reported in the clinical file of an older man with visual impairments: “He receives 2.5 h of direct allocation per week to help with household chores. His wife does the dusting” [File 02]. However, this social worker mentioned the risk of overcompensation in such context: “He is in a residence where everything is paid for and anticipated by the person in charge, which is not bad in itself but it doesn’t help people maintain their capacities or autonomy” [H06]. For this caregiver, who lived with her husband, the needs for transport were generally satisfied: “For my groceries, I have my employee who has her own car. If we need to go to the doctor or go shopping for something, we use paratransit” [C02].

Moreover, needs for social activities were rarely fulfilled, especially for activities of community life and interpersonal relationships domains of participation (Table 4). Nevertheless, a median of five participants had fulfilled needs in the responsibilities domain. Hence, most needs related to personal and financial responsibilities were fulfilled by caregivers as mentioned by this woman who assumed her husband’s personal choices: “We don’t share [decisions] like we used to. I make all the decisions, I take charge of everything” [C08]. In that other case, the older man’s needs to go shopping were compensated through respite periods, which allowed his wife to realize this important activity in community: “If I don’t have time to get groceries and run errands during the week, I do it those days. And I take a couple of hours to go shopping for him” [C07]. Finally, this older man described having relaxing activities, which he did mostly by himself at home: “I love to read and watch television. I also have my flowers, which I water. I have a green thumb” [A05]. However, needs to participate in leisure were generally more rarely satisfied.

### Unmet needs

Older adults’ unmet needs mainly involved leisure, fitness, community life, interpersonal relationships and mobility (Table 4). First, for participation in daily activities, fitness and mobility needs were mostly unmet, respectively for a median of nine and seven older adults. Specific support was needed to help older adults have a healthier diet, as reported by this caregiver: “We would have liked to have somebody to prescribe—to make her, even though you cannot make someone—prescribe something to help her eat less” [C09]. The HSSCs often do not have nutritionist personnel. Therefore, needs related to healthy eating were mainly unmet, as this nurse explained: “Patients are not all seen by dietitians; we see some with problems swallowing and some who are undernourished, who eat very little and lose a lot of weight” [H09]. This other older woman expressed an important unmet need about going to the restaurant, due to limited accessibility of the environment: “What I would ask for would be to have more [help] to go eat at the restaurant once or twice a week” [A01].Sleeping problems were generally identified, as this caregiver acknowledged in regards to her husband’s complex breathing problems: “He always had trouble sleeping. But now he sleeps during the day. He wakes up often in the night. He has sleep apnea. He went to see someone. They tried a device at night but he wasn’t able [to breathe]. So he doesn’t have anything” [C10]. Needs to perform physical exercises in order to keep as fit as possible were generally unsatisfied. For example, this older man, interested in cycling, expressed needs for some adaptations considering his visual impairment: “I’d like to go bike riding in the summer. At my own speed… Maybe I could get a three-wheeled bike” [A02]. Cognitive stimulation, important for the older adults and their caregivers, remained unmet, as mentioned by this caregiver whose mother had memory problems: “Sometimes she can call me five or six times about the following week, until it’s over. It’s harassing, sometimes. I know she’s a bit bored and I can see that it bothers her” [C05]. Another caregiver identified unmet needs related to the older woman’s difficulty expressing her needs: “If she were more stimulated in communication. But for the moment I can’t think of any service that offers that” [C11]. This older man explained how going outside was a challenge mostly because of his physical conditions: “I’m unable to walk backyards anymore with the operations I had. I’d take regular walks, but I won’t walk unnecessarily unless I get lost. I plan where I want to go so I walk as little as possible” [A02].

Then, many of the unmet needs were about social activities (Table 4), particularly regarding leisure and community life that concerned, respectively, a median of ten and nine older adults. Needs about family relationships and friendships were most of the time unfulfilled. As recognized by this caregiver, family relationships were often limited: “We live to be too old [laughter]. So both of us are mostly alone, the family is not big” [C02]. This other older adult described the difficulty to have opportunities for and to be involved in his community: “I’d love to go but no one [that I know] wants to organize anything. We are too old to do it” [A04]. This son explained that having more social contacts and doing group activities could help his mother, but such needs were rarely fulfilled: “Perhaps being in more contact with other people would help her want to get better or feel better. More contacts, something to get her out of the house […], something that makes her want to live rather than suffering from aging and boredom” [C11]. Others, such as this older man, described the challenge of having unmet needs in sports activities: “I feel… I feel stuck. […] A prisoner. Because, before, we used to go swimming and all that, but now neither of us can do anything” [A11]. Interestingly, the mental healthcare provider gave specific attention to unmet needs about leisure activities: “What dissatisfies him the most is that there aren’t any activities at the residence. There are no social, fun or recreational activities” [H06].

## Discussion

The main purpose of this study was to explore needs related to participation in daily and social activities of older adults having disabilities and receiving home care services from the perspective of the older adults, their caregivers and healthcare providers. The results of this study add new insights to other studies done with older adults with functional decline and living at home, which mainly focused on unmet needs in daily activities [26, 29] and did not necessarily address social activities. Findings indicated that the perceived needs were identified in all domains of participation, including in basic daily but also more complex social activities. Since successful aging in place involves multiple actors with diverse perspectives [40], these findings highlight the complexity of older adults’ participation needs and the challenge of properly allocating associated resources [41]. Results are similar to those of one study conducted among healthy older adults which found that nearly all domains of participation were increasingly restricted with age [4]. Those mostly restricted were personal care, housing, mobility, as well as community life and leisure activities. In our study, most perceived needs were also related to these latter domains. Other domains such as fitness, nutrition, responsibilities and interpersonal relationships were somewhat not found to diminish with normal aging [4], while in our study, they were a specific challenge for most of the older adults having disabilities. Therefore, perceived needs that have been identified could be partially explained by aging, but also by the presence of disability. Moreover, a study carried out with people who had a stroke found that participation in daily and social activities, except for the interpersonal relationship domain, was significantly more reduced when compared to normal aging [42]. In addition, restrictions in participation were found generally greater in social activities of older adults with visual impairments compared to those without such problems [43]. This further restriction in social activities compared to daily activities was also observed with people having mental illness [44], mild cognitive problems [45] and other physical disabilities [18]. Since older adults aspire to remain socially active throughout aging, despite having disabilities, social and healthcare systems must find innovative ways to reduce social and environmental barriers to their participation.

Many needs revealed in our study involved activities ‘outside’ the house, such as going to the restaurant, walking outside, or being involved in the community. Considering that these more complex activities involve mainly environmental barriers, population health initiatives are being implemented that focus on such factors. For example, the World Health Organization [46] developed the Global Network of Age-friendly Cities, an important initiative that promotes the importance of maintaining and improving older adults’ participation, no matter their level of capacities. Concretely, this initiative is engaged in informing about activities and existing services, and also integrating new activities adapted to people with disabilities. By encouraging accessible community environments that promote safety, mobility, and flexible and affordable means of transportation, the Age-friendly Cities initiative promotes collaboration and partnership between multiple sectors of local communities. Therefore, as the perceived participation needs of the older adults in our study were explained not just by disabilities but also by aging, such innovations might be particularly interesting.

To promote and improve participation of older adults having disabilities, individual and personalized interventions might be needed to complement such population health initiatives. One example of these initiatives, the Personalized Attendant for Community Integration (*Accompagnement Personnalisé d’Intégration Communautaire*; APIC), consists of a community follow-up conducted by a trained citizen [47]. The attendant is trained to meet with the older adult three hours per week for a year to offer assistance in accomplishing the person’s projects and provide education to adapt activities he/she finds meaningful [48]. This intervention aims to support the social integration and participation of adults living with disabilities, and is currently being adapted for older adults having disabilities. Among other examples, the Lifestyle Redesign*®* intervention [49, 50] was developed to enhance the health and well-being of community-dwelling older adults through the design of health-promoting and balanced daily routines, as well as participation in meaningful activities. This group and individual intervention stimulates social integration and addresses different themes relevant to older adults (occupations; aging, health and occupation; transportation; safety; social relationships; cultural awareness; and finances). As this intervention is currently being translated into French and adapted for Québec, more research is needed before putting these innovative interventions into practice.

Among the domains of participation, the needs in daily activities, such as personal care, nutrition and housing, were mainly fulfilled, but social activities were more rarely. Such discrepancies question the capacity of HSSCs to identify and fulfill the whole range of the participation needs of older adults having disabilities, including for social activities, leisure and community life. Currently mainly based on the SMAF [31], home healthcare providers’ assessment of clients’ needs focus principally on daily activities [51]. Our data pinpoints that the needs the most completely fulfilled include meal preparation, groceries, taking medication and home maintenance. Such focus on basic and urgent in-home activities might bring to an emergency practice context [52] and oppose to long-term care principles [53]. These principles involve adopting a community approach within the living environments and are especially relevant with people having chronic health problems and disabilities [54]. Moreover, it has been shown that priorities established by healthcare providers mainly emphasize the technical management of diseases, while patients’ priorities concern comprehensive care, including self-care support, participation in clinical decisions, and partnership with community organizations [55]*.* The qualitative findings in this study combined the older adults’ perspectives with those of the caregivers and healthcare providers and sought to not underestimate unmet needs, which might be less specific to the fulfilled ones. Given that 90 % of needs are fulfilled by informal caregivers [56], who are especially important for older adults’ participation in social activities [29], it is essential to involve them in needs assessment [53]. The findings of our study regarding the most fulfilled participation needs reveal the importance of assessing needs more broadly and in further details and of paying specific attention to unmet ones.

Resources currently offered to older adults having disabilities might not optimally target activities that are most associated with health and well-being [18, 57]. Although identified equally in both daily and social activities, needs were mainly unmet in social activities, mostly leisure, interpersonal relationships and community life, and fewer daily activities such as mobility and fitness. These results are consistent with those of two previous studies conducted among stroke survivors which found that most unmet needs are those related to psychological and social aspects, such as community life and leisure [27, 58]. Both daily and social activities need to be realized in a person’s routine to contribute to health and well-being [59]. For example, having a good sleep is closely related to the level of energy available for participation [57] and, in turn, active leisure, group activities or physical exercises can improve quality of sleep [60]. Given the well-demonstrated benefits of fitness and participation in social activities [57], the older adult’s unmet needs in these domains are worrisome. Possible benefits of such active lifestyles also include reduced mortality [61, 62], slower cognitive decline [62], decreased drug use, reduced use of health services [63], and reduced depressive symptoms [64]. As older adults in our study had difficulties in expressing their needs, considering meaning, level of interest and importance of activities could allow a better identification of unmet needs and improving quality of life [65]. Activities such as leisure or fitness are mostly realized for the person’s own sake and cannot be delegated or compensated without losing the benefit from them [61]; they allow older adults to not only add years to their lives, but also life to their years. Previous studies also uncovered the importance of meaningful activities and well-being for older adults living at home [29]. Finally, by improving clients’ empowerment [66, 67], providing client-centred needs assessment [51, 65], and developing partnerships with community organizations, older adults’ social needs could be better met.

### Implications for practice, research and policy

Considering HSSCs’ legal obligation with their local partners to promote population health and well-being, including helping older adults having disabilities to participate in the community [20], it is important to assess and meet needs of the persons among their territory, even those who do not actively ask for services. Acknowledging the importance of fully assessing needs for participation in daily and social activities could help HSSCs and healthcare providers to better integrate health-promoting practices and adopt a preventive approach. To do so, preventive home visits [68] and group interventions [50] might be particularly helpful in meeting the needs of older adults having disabilities and ultimately enhancing population health and well-being. In addition, older adults’ unmet needs and expectations regarding their optimal participation in society [15, 54] have to be targeted by the community resources, including the HSSC healthcare providers, community organizations and private enterprises. Accordingly, several interventions are known to be innovative and efficacious in increasing older adults and caregivers’ empowerment in taking care of them independently [66, 67]. Exploring meaning, interests and importance of activities should be undertaken by healthcare providers through their assessment. Participatory action research could be an interesting option to accompany such a change in practice [69].

### Strengths and limitations

To our knowledge, this study is the first to provide an in-depth qualitative exploration of older adults’ participation needs from their perspective and to merge these perceptions with those of their caregivers and healthcare providers. Rigour was ensured through extensive data collection, constant monitoring of analysis and interpretations, as well as validation of findings with participants following the interviews [37]. As with other qualitative studies, results are time- and context-sensitive and influenced by the researchers, which were limited by in-depth description of participants’ characteristics and contexts. Triangulated sources (semi-structured interviews, sociodemographic questionnaires, reviews of clinical record) and sample diversification were used to offset further limitation [34]. Among its limitations, this study included only one HSSC, although typical of such health organizations in Québec, and a limited number of older adults, which all had a caregiver and were known by a healthcare provider. Although employment and education are essential basic rights of people having disabilities, these domains were not considered for the current analysis, since they were not mentioned by the study participants; however, they should be addressed in future studies. Finally, reassuring participants that there were no right or wrong answers minimized social desirability, a potential bias [34].

## Conclusions

This paper provides insights about needs for participation in daily and social activities of older adults having disabilities and living at home, as perceived by themselves, their caregivers and their healthcare providers. Our findings show that perceived needs related to all domains of participation. Daily activities, such as personal care, nutrition and housing, and some social activities, such as managing a budget and going shopping, were generally fulfilled. Unmet needs mainly concerned activities most associated with health and well-being, including leisure, community life, fitness, interpersonal relationships and mobility. To help older adults age in place and be actively engaged with life, healthcare providers must acknowledge the complexity of participation needs and consider people’s unique experiences related to being at home and to their participation in the community. Involving caregivers and clients as partners for needs assessment is essential to produce a more complete needs assessment and reveal comprehensive met and unmet needs. Moreover, considering meaning, interest and importance of activities through a client-centred assessment and developing partnerships with community organizations could help resources to optimally target activities that are most associated with health and well-being. To better integrate resources and meet older adults’ needs, recognizing the contribution of each type of service provider, including community organizations as well as private resources, would also be helpful. Further research is needed to analyze needs related to employment and education and to examine in more depth discrepancies between different actors’ perceptions about older adults’ participation needs.

## Abbreviations

HSSC: Health and Social Services Centre
